# A Molecular Phylogenomic Analysis of the ILR1-Like Family of IAA Amidohydrolase Genes

**DOI:** 10.1002/cfg.340

**Published:** 2003-12

**Authors:** James J. Campanella, Daniel Larko, John Smalley

**Affiliations:** 1 Montclair State University Department of Biology and Molecular Biology 1 Normal Avenue Montclair NJ 07043 USA; 2 Bergen Community College Department of Science and Technology 400 Paramus Road Paramus NJ 07652 USA

## Abstract

The ILR1-like family of hydrolase genes was initially isolated in *Arabidopsis thaliana* and is thought to help regulate levels of free indole-3-acetic-acid.We have investigated how this family has evolved in dicotyledon, monocotyledon and gymnosperm species by employing the GenBank and TIGR databases to retrieve orthologous genes. The relationships among these sequences were assessed employing phylogenomic analyses to examine molecular evolution and phylogeny. The members of the ILR1-like family analysed were ILL1, ILL2, ILL3, ILL6, ILR1 and IAR3. Present evidence suggests that IAR3 has undergone the least evolution and is most conserved. This conclusion is based on IAR3 having the largest number of total interspecific orthologues, orthologous species and unique orthologues. Although less conserved than IAR3, DNA and protein sequence analyses of ILL1 and ILR1 suggest high conservation. Based on this conservation, IAR3, ILL1 and ILR1 may have had major roles in the physiological evolution of ‘higher’ plants. ILL3 is least conserved, with the fewest orthologous species and orthologues. The monocotyledonous orthologues for most family-members examined have evolved into two separate molecular clades from dicotyledons, indicating active evolutionary change. The monocotyledon clades are: (a) those possessing a putative endoplasmic reticulum localizing signal; and (b) those that are putative cytoplasmic hydrolases. IAR3, ILL1 and ILL6 are all highly orthologous to a gene in the gymnosperm *Pinus taeda*, indicating an ancient enzymatic activity. No orthologues could be detected in *Chlamydomonas*, moss and fern databases.


**Comparative and Functional Genomics**
*Comp Funct Genom* 2003; **4**: 584–600.
Published online in Wiley InterScience (www.interscience.wiley.com). **DOI:** 10.1002/cfg.340

**Primary Research Paper**

### A molecular phylogenomic analysis of the ILR1-like family of IAA amidohydrolase genes

James J. Campanella[1]*, Daniel Larko[1] and John Smalley[2]
[1]*Montclair State University, Department of Biology and Molecular Biology, 1 Normal Avenue, Montclair, NJ 07043, USA*
[2]*Bergen Community College, Department of Science and Technology, 400 Paramus Road, Paramus, NJ 07652, USA*

**Correspondence to: James J. Campanella, Montclair State University, Department of Biology and Molecular Biology, 1 Normal Avenue, Montclair, NJ 07043, USA. E-mail: james.campanella@montclair.edu*

Received: 6 May 2003
Revised: 17 September 2003
Accepted: 30 September 2003

### Abstract

**The ILR1-like family of hydrolase genes was initially isolated in *Arabidopsis thaliana* and is thought to help regulate levels of free indole-3-acetic-acid. We have investigated how this family has evolved in dicotyledon, monocotyledon and gymnosperm species by employing the GenBank and TIGR databases to retrieve orthologous genes. The relationships among these sequences were assessed employing phylogenomic analyses to examine molecular evolution and phylogeny. The members of the ILR1-like family analysed were ILL1, ILL2, ILL3, ILL6, ILR1 and IAR3. Present evidence suggests that IAR3 has undergone the least evolution and is most conserved. This conclusion is based on IAR3 having the largest number of total interspecific orthologues, orthologous species and unique orthologues. Although less conserved than IAR3, DNA and protein sequence analyses of ILL1 and ILR1 suggest high conservation. Based on this conservation, IAR3, ILL1 and ILR1 may have had major roles in the physiological evolution of 'higher' plants. ILL3 is least conserved, with the fewest orthologous species and orthologues. The monocotyledonous orthologues for most family-members examined have evolved into two separate molecular clades from dicotyledons, indicating active evolutionary change. The monocotyledon clades are: (a) those possessing a putative endoplasmic reticulum localizing signal; and (b) those that are putative cytoplasmic hydrolases. IAR3, ILL1 and ILL6 are all highly orthologous to a gene in the gymnosperm *Pinus taeda*, indicating an ancient enzymatic activity. No orthologues could be detected in *Chlamydomonas*, moss and fern databases. Copyright © 2003 John Wiley & Sons, Ltd.**

**Keywords: phylogenetics; phylogenomics; ILR1-like family; *Arabidopsis*; auxin regulation; IAA amidohydrolase**

### Introduction

The ILR1-like IAA amidohydrolase family helps regulate free indole-3-acetic acid (IAA or auxin) concentrations in *Arabidopsis thaliana* (Bartel and Fink, 1995; Davies *et al.*, 1999; LeClere *et al.*, 2002; Cooke *et al.*, 2002). In 'higher' plants, IAA stimulates gene expression, cell division, cell elongation and differentiation in plant tissue. The hormone is stored in an inactive, conjugated form and can be found in two types: (a) in dicotyledons, as an amide-linked IAA form bound to an amino acid; and (b) in monocotyledons, as an ester-linked form bound to a sugar (Bandurski *et al.*, 1969; Cohen and Bandurski, 1982; Domagalski *et al.*, 1987). On average, 95% of all IAA in a plant is conjugated into this non-stimulating form.

Amide conjugates account for the bulk of conjugated IAA in the dicotyledons. IAA-alanine, IAA-aspartate, IAA-leucine and IAA-glutamate have been detected in *Arabidopsis thaliana* (Tam *et al.*, 2000; Kowalczyk and Sandberg, 2001). IAA-aspartate and IAA-glutamate have been identified as natural conjugates in cucumber (Sonner and Purvis, 1985) and soybean (Cohen, 1982). IAA-alanine has been detected in *Picea abies* (Ostin *et al.*, 1992).

Common IAA-sugar moieties in monocotyledons include IAA-inositol, IAA-myo-inositol, and

Copyright © 2003 John Wiley & Sons, Ltd.

IAA-glucose (Bandurski *et al.*, 1969; Kopcewicz *et al.*, 1974; Bandurski and Schulze, 1977; Campanella *et al.*, 1996). The primary auxin conjugates of gymnosperms seem to be ester-linked to sugars (Sandberg, 1984; Ljung *et al.*, 2001), although purely amide-linked conjugates have been found (Andersson and Sandberg, 1982).

Several IAA amidohydrolases (ILR1, IAR3, ILL1, ILL2, ILL3, ILL5 and ILL6), known collectively as the ILR1-like family, have been isolated from *Arabidopsis thaliana* (Bartel and Fink, 1995; LeClere *et al.*, 2002). Another ILR1-like family member, sILR1, has also been isolated from the closely related species *Arabidopsis suecica* (Campanella *et al.*, 2003a).

In this study, we employed comprehensive searches of online databases to determine the genomic phylogeny of amidohydrolases in the ILR1-like family of genes, employing them as a model to study molecular evolution and change among various dicotyledons, monocotyledons and gymnosperms. Our studies were designed to examine how genes change over time between species that are closely or distantly related, as well as to give us an indication of how significant certain gene families are in gymnosperms and angiosperms.

Employing online databases, we have identified 15 species having DNA and protein sequences orthologous to the *Arabidopsis* sILR1/ILR1, ILL1, ILL2, ILL3, ILL6 and IAR3 genes. We have examined genomes from the following species: lettuce (*Lactuca sativa*); ice plant (*Mesembryanthemum crystallinum*); loblolly pine (*Pinus taeda*); grape (*Vitis vinifera*); cotton (*Gossypium hirsutum*); barrel clover (*Medicago truncatula*); soybean (*Glycine max*); barley (*Hordeum vulgare*); tomato (*Lycopersicum esculentum*); potato (*Solanum tuberosum*); corn (*Zea mays*); sorghum (*Sorghum bicolor*); lotus (*Lotus japonicus*); wheat (*Triticum aestivum*); and rice (*Oryza sativa*). All these species contain orthologues to members of the ILR1-like family. We have attempted here to demonstrate the value of these sequences to the study of molecular evolution of gene families.

### Materials and methods

### Sources of sequence data

All non-*Arabidopsis* sequences were obtained from incomplete genome projects, partial expression sequence tags (ESTs) or cDNA clones and, as such, are not comprehensive. DNA orthologue searches were conducted using the resident BLAST search engine on the TIGR website (**http://tigrblast.tigr.org/tgi**; Altschul *et al.*, 1997). On all searches, the cut-off BLAST score for orthologue inclusion was 1000, to ensure a highly stringent analysis with statistically high confidence values for functionality. Additionally, we noted that orthologous sequences at BLAST scores of less than 1000 were, more often than not, incomplete ESTs.

The GenBank ILR1 cDNA sequences used for orthologue searches were *A. thaliana* (GenBank Accession No. At3g02875) and *A. suecica* (GenBank Accession No. AF468012). Homologous ILR1 sequences found on TIGR included: loblolly pine (*P. taeda*) (TC3794); corn (*Z. mays*) (TC113408, TC180315, TC187808); barley (*H. vulgare*) (TC36547, TC78880); wheat (*T. aestivum*) (TC48711, TC55038, TC49296, TC88968); sorghum (*S. bicolor*) (TC42893); tomato (*L. esculentum*) (TC107732, TC116615, TC119522); potato (*S. tuberosum*) (BG598945, TC67969, TC59567); grape (*V. vinifera*) (TC8799, TC12056); lotus (*L. japonicus*) (TC4684); lettuce (*L. sativa*) (BQ861729); barrel clover (*M. truncatula*) (TC77458, TC77457, TC87843, TC80526, TC89442); rice (*O. sativa*) (NP657299, TC115841, TC126427); and soybean (*G. max*) (TC160462, TC160463, TC161567).

The *A. thaliana* sequence IAR3 (GenBank Accession No. At1g51760) was employed, with introns removed by hand, to search for orthologues. Homologous IAR3 sequences found on TIGR included: loblolly pine (*P. taeda*) (TC15163); barrel clover (*M. truncatula*) (BE123972, TC80526, TC79815, TC87843, TC89442, TC77457, TC-77458); lotus (*L. japonicus*) (TC966, AW719 214); wheat (*T. aestivum*) (TC88968, TC95398); rice (*O. sativa*) (TC124940, TC118919, TC126427, TC127427); soybean (*G. max*) (TC120817, TC121-750, TC160463, TC160465); grape (*V. vinifera*) (TC12056, TC8799, CB912650); ice plant (*M. crystallinum*) (AW266 116); tomato (*L. esculentum*) (TC105095, TC99719); sorghum (*S. bicolor*) (TC47382); cotton (*G. hirsutum*) (BF278001); lettuce (*L. sativa*) (BQ871198); corn (*Z. mays*) (TC180315, TC183440); and potato (*S. tuberosum*) (TC50170, TC50186, TC47432, TC72910). One barley (*H. vulgare*) (BF261986) orthologue was

Copyright © 2003 John Wiley & Sons, Ltd. 

found on the GenBank database with a second from TIGR (TC78880).

The *A. thaliana* cDNA sequence for ILL1 (GenBank Accession No. AtU23 795) was employed to search for orthologues using the TIGR BLAST engine. Homologous ILL1 sequences found on TIGR included: loblolly pine (*P. taeda*) (TC15163); barrel clover (*M. truncatula*) (TC77458, TC77457, TC80526, TC87843, TC79815); lotus (*L. japonicus*) (TC4684); wheat (*T. aestivum*) (TC88968, TC64060); rice (*O. sativa*) (TC124940, TC118919, TC127427, NP65-7299, TC126427); soybean (*G. max*) (TC160462, TC160463, TC161567, TC160465); grape (*V. vinifera*) (TC12056, TC8799); tomato (*L. esculentum*) (TC116615, TC119522, TC120112); sorghum (*S. bicolor*) (TC47337); cotton (*G. hirsutum*) (BF278001); lettuce (*L. sativa*) (BQ871198); corn (*Z. mays*) (TC183440, TC183315); barley (*H. vulgare*) (TC78880); and potato (*S. tuberosum*) (TC59567, TC67969, TC72910).

The *A. thaliana* cDNA sequence for ILL2 (GenBank Accession No. At5g56660) was employed to search for orthologues. Homologous ILL2 sequences found on TIGR included: barrel clover (*M. truncatula*) (TC77458, TC77457, TC87843, TC80526, TC79815); lotus (*L. japonicus*) (TC4684); wheat (*T. aestivum*) (TC88968, TC95398, TC64060, TC63551); rice (*O. sativa*) (TC127427, TC124940, NP657299, TC115841, TC126427); soybean (*G. max*) (TC160462, TC160-463, TC161567); grape (*V. vinifera*) (TC8799, TC12056); tomato (*L. esculentum*) (TC116615, TC119522); sorghum (*S. bicolor*) (TC47337); lettuce (*L. sativa*) (BQ861729); corn (*Z. mays*) (TC183440, TC180315, TC187808); barley (*H. vulgare*) (TC78880); and potato (*S. tuberosum*) (TC59567, TC67969, TC64521).

The *A. thaliana* cDNA sequence for ILL6 (GenBank Accession No. AY065 996) was employed to search for orthologues. Homologous ILL6 sequences found on TIGR included: loblolly pine (*P. taeda*) (TC15163); barrel clover (*M. truncatula*) (TC77458, TC89442); lotus (*L. japonicus*) (TC46844); wheat (*T. aestivum*) (TC88968); rice (*O. sativa*) (TC124940); soybean (*G. max*) (TC161567, TC160463); grape (*V. vinifera*) (TC12056, TC8799); tomato (*L. esculentum*) (TC120112, TC116615); corn (*Z. mays*) (TC180-315); barley (*H. vulgare*) (TC78880); and potato (*S. tuberosum*) (TC71140, TC59567).

Finally, the *A. thaliana* ILL3 DNA sequence (GenBank Accession No. At5g54140) was employed to search for orthologues. ILL3 introns were removed by hand to leave the cDNA of the primary coding sequence for the search. Homologous ILL3 sequences found on TIGR included: cotton (*G. hirsutum*) (Accession No. TC11274); barrel clover (*M. truncatula*) (TC87843, TC87458, TC77457); wheat (*T. aestivum*) (TC45334); soybean (*G. max*) (TC140147); tomato (*L. esculentum*) (TC107087); and corn (*Z. mays*) (TC136446, AY108 775).

Protein sequences were generated by first determining the correct reading frame of each EST using the GeneMark ORF-finding program (**http://opal.biology.gatech.edu/GeneMark/**; M. Borodovsky and A. Lukashin, Georgia Tech University). The 5′ and 3′ untranslated regions of each EST were removed by hand, and the DNA sequences translated to proteins by application of the Transeq program (Rice *et al*., 2000).

The ILL1, ILL2, ILL3, ILL6, ILR1 and IAR3 cDNA and protein sequences were also used to search moss and fern databases. As with the other searches, the cDNA of the primary coding sequence was employed.

### Sequence alignments and phylogenetic tree construction

Multiple alignments of ILR1 and IAR3 DNA and amino acid orthologues were constructed using the CLUSTAL X version 1.8 software (Thompson *et al*., 1997). The multiple parameters adopted for IAR3, ILL1 and ILL2 protein alignments were 'Opening 70, Extension 0.75'. The IAR3 DNA alignments were performed using parameters of 'Opening 75' and 'Extension 12'. All other alignment settings were employed at default values. The DNA and protein sequences of a bacterial M20 peptidase from *Campylobacter jejuni* (GenBank Accession No. Z36 940) were used as outgroups in all studies.

Phylogenetic trees were generated from the distances provided by the CLUSTAL X analysis using the neighbour-joining method (Saitou and Nei, 1987).

Bootstrap analyses (Felsenstein, 1985) consisted of 1000 replicates using the same protocol. The neighbour-joining trees were visualized with the TREEVIEW program (Page, 1996). All bootstrap values that are less than 500 are not shown on phylograms.

Copyright © 2003 John Wiley & Sons, Ltd.

### Results

### ILR1-like family orthologue analysis

An initial correlative analysis of the orthologues was performed (Table 1). The analysis indicates that a large number of the orthologues are specifically homologous only for certain members of the ILR1-like family; of the 66 total orthologues found in TIGR and GenBank, 30 appear to be homologous to only a single ILR1-like family member. Of the orthologues, 40% (27 of 66) were either partly sequenced ESTs (19) or putative pseudogenes (8) with early stop codons (Table 1). Of the full-length orthologues, 30% (12 of 39) contained a putative endoplasmic reticulum (ER) localization signal, as do ILR1, ILL1, ILL2 and IAR3 (Table 1, bold). The remaining orthologues are putatively cytoplasmically localized, since no obvious localization signal was detectable, as in ILL3 and ILL6.

The ILR1 primary coding sequence appears to have diverged based on the phylogenetic separation developed between monocotyledons and dicotyledons in both the DNA and protein phylograms (Figure 1). All the monocotyledons (bold), except rice (TC126427), fall together into two large clades

**Table 1.** Orthologous ILR1-like genes detected in the various species examined

| Species | Gene locus | Orthologous to ILR1-like family member |
|---|---|---|
| ***G. max*** | TC160462 | ILL1, ILL2, ILR1 |
| *G. max* | TC160463 | ILL2, ILL6, ILR1, IAR3 |
| *G. max* | TC161567 | ILL1, ILL2, ILR1 |
| *G. max* (incomplete) | TC160465 | ILL1, IAR3 |
| *G. max* (pseudogene) | TC140147 | ILL3 |
| ***G. max*** | TC120817 | IAR3 |
| *G. max* | TC121750 | IAR3 |
| *G. hirsutum* | BF278001 | ILL1, IAR3 |
| *G. hirsutum* (incomplete) | TC11274 | ILL3 |
| ***H. vulgare*** | TC78880 | ILL1, ILL2, ILL6, ILR1, IAR3 |
| *H. vulgare* (incomplete) | TC36547 | ILR1 |
| ***H. vulgare*** | BF261986 | IAR3 |
| *L. Japonicus* (incomplete) | TC4684 | ILL1, ILL2, ILL6, ILR1, IAR3 |
| *L. japonicus* (incomplete) | AW719214 | IAR3 |
| *L. sativa* (pseudogene) | BQ871198 | ILL1, IAR3 |
| *L. sativa* (incomplete) | BQ861729 | ILL2, ILR1 |
| *L. esculentum* | TC119522 | ILL1, ILL2, ILR1 |
| ***L. esculentum*** | TC116615 | ILL2, ILL6, ILR1 |
| *L. esculentum* | TC107087 | ILL3 |
| *L. esculentum* (incomplete) | TC120112 | ILL6 |
| *L. esculentum* | TC105095 | IAR3 |
| ***L. esculentum*** | TC99719 | IAR3 |
| *M. crystallinum* (pseudogene) | AW266116 | IAR3 |
| ***M. truncatula*** | TC77457 | ILL1, ILL2, ILL3, ILR1, IAR3 |
| ***M. truncatula*** | TC77458 | ILL1, ILL2, ILL3, ILR1, IAR3 |
| *M. truncatula* (pseudogene) | TC80526 | ILL1, ILL2, ILR1, IAR3 |
| *M. truncatula* | TC87843 | ILL1, ILL2, ILL3, ILR1, IAR3 |
| *M. truncatula* | TC79815 | ILL1, ILL2, IAR3 |
| *M. truncatula* (pseudogene) | TC89442 | ILL6, ILR1, IAR3 |
| *M. truncatula* | BE123972 | IAR3 |
| ***O. sativa*** | TC124940 | ILL1, ILL2, ILL6, ILR1, IAR3 |
| ***O. sativa*** | TC118919 | ILL1, IAR3 |
| *O. sativa* | NP657299 | ILL1, ILL2, ILR1 |
| *O. sativa* | TC126427 | ILL1, ILL2, ILR1, IAR3 |
| *O. sativa* | TC127427 | ILL1, ILL2, IAR3 |
| *O. sativa* | TC115841 | ILL2, ILR1 |
| *O. sativa* (pseudogene) | TC91825 | ILR1 |

Copyright © 2003 John Wiley & Sons, Ltd. 

**Table 1.** Continued

| Species | Gene locus | Orthologous to ILR1-like family member |
|---|---|---|
| *P. taeda* (incomplete) | TC15163 | ILL1, ILL6, IAR3 |
| *S. bicolor* | TC47382 | ILL1, ILL2, IAR3 |
| *S. bicolor* | TC42893 | ILR1 |
| *S. tuberosum* (incomplete) | TC59567 | ILL1, ILL2, ILL6, ILR1 |
| *S. tuberosum* | TC67969 | ILL1, ILL2, ILR1 |
| *S. tuberosum* (incomplete) | TC72910 | ILL1, IAR3 |
| *S. tuberosum* (incomplete) | TC64521 | ILL2 |
| *S. tuberosum* (pseudogene) | TC71140 | ILL6 |
| *S. tuberosum* (incomplete) | BG598945 | ILR1 |
| *S. tuberosum* | TC50170 | IAR3 |
| *S. tuberosum* | TC50186 | IAR3 |
| *S. tuberosum* | TC47432 | IAR3 |
| ***T. aestivum*** | TC88968 | ILL1, ILL2, ILL6, ILR1, IAR3 |
| *T. aestivum* (incomplete) | TC64060 | ILL1, ILL2 |
| *T. aestivum* | TC95398 | ILL2, IAR3 |
| *T. aestivum* (pseudogene) | TC63551 | ILL2 |
| *T. aestivum* (incomplete) | TC45334 | ILL3 |
| *T. aestivum* | TC48711 | ILR1 |
| *T. aestivum* (incomplete) | TC55038 | ILR1 |
| *T. aestivum* (incomplete) | TC49296 | ILR1 |
| *V. vinifera* (incomplete) | TC12056 | ILL1, ILL6, ILR1, IAR3 |
| *V. vinifera* | TC8799 | ILL1, ILL2, ILL6, ILR1, IAR3 |
| *V. vinifera* (incomplete) | CB912650 | IAR3 |
| ***Z. mays*** | TC180315 | ILL1, ILL2, ILL6, ILR1, IAR3 |
| *Z. mays* | TC183440 | ILL1, ILL2, IAR3 |
| *Z. mays* | TC187808 | ILL2, ILR1 |
| *Z. mays* (incomplete) | TC136446 | ILL3 |
| *Z. mays* | AY108775 | ILL3 |
| *Z. mays* | TC113408 | ILR1 |

Gene products in bold contain an ER localization signal. Putative incomplete ESTs and pseudogenes are indicated.

(Figure 1A). The presence of the two monocotyledon clades suggests active evolutionary change within these species. The strength of the monocotyledon DNA analysis is indicated by the bootstrap scores of 100% and 89.7% (bold) on the branches of the monocotyledon clades (Figure 1A). Closer examination of the two clades indicates a molecular separation based on the presence of a putative ER localization signal (Figure 1) All those members of the smaller clade possess a putative signal while those in the larger clade appear to be cytoplasmically localized.

Except for some minor branch rearrangements, there is little change between the topology of the ILR1 protein and DNA phylograms (Figure 1). The two trees do not differ much in placement or structure of the various orthologues and paralogues.

Potato (TC67969, TC59567) and tomato (TC116-615, TC119522) fall into the same clade in both the DNA and protein neighbour-joining trees of ILR1 orthologues (Figure 1), indicating both DNA and amino acid sequence conservation. Since these two species are both in the Solanaceae family, it is expected that they are phylogenomically similar, but another potato orthologue (BG598945) does not join either clade.

The IAR3 phylograms (Figure 2) are the most highly branched of any of the ILR1-like family members. Using our highly stringent assay conditions, IAR3 possesses more orthologous species (15) than any other family member. In addition, searches with the IAR3 sequence produce more orthologues (36) than the other ILR1-like sequences. These two observations suggest that IAR3 is highly conserved, since the interspecific

Copyright © 2003 John Wiley & Sons, Ltd.

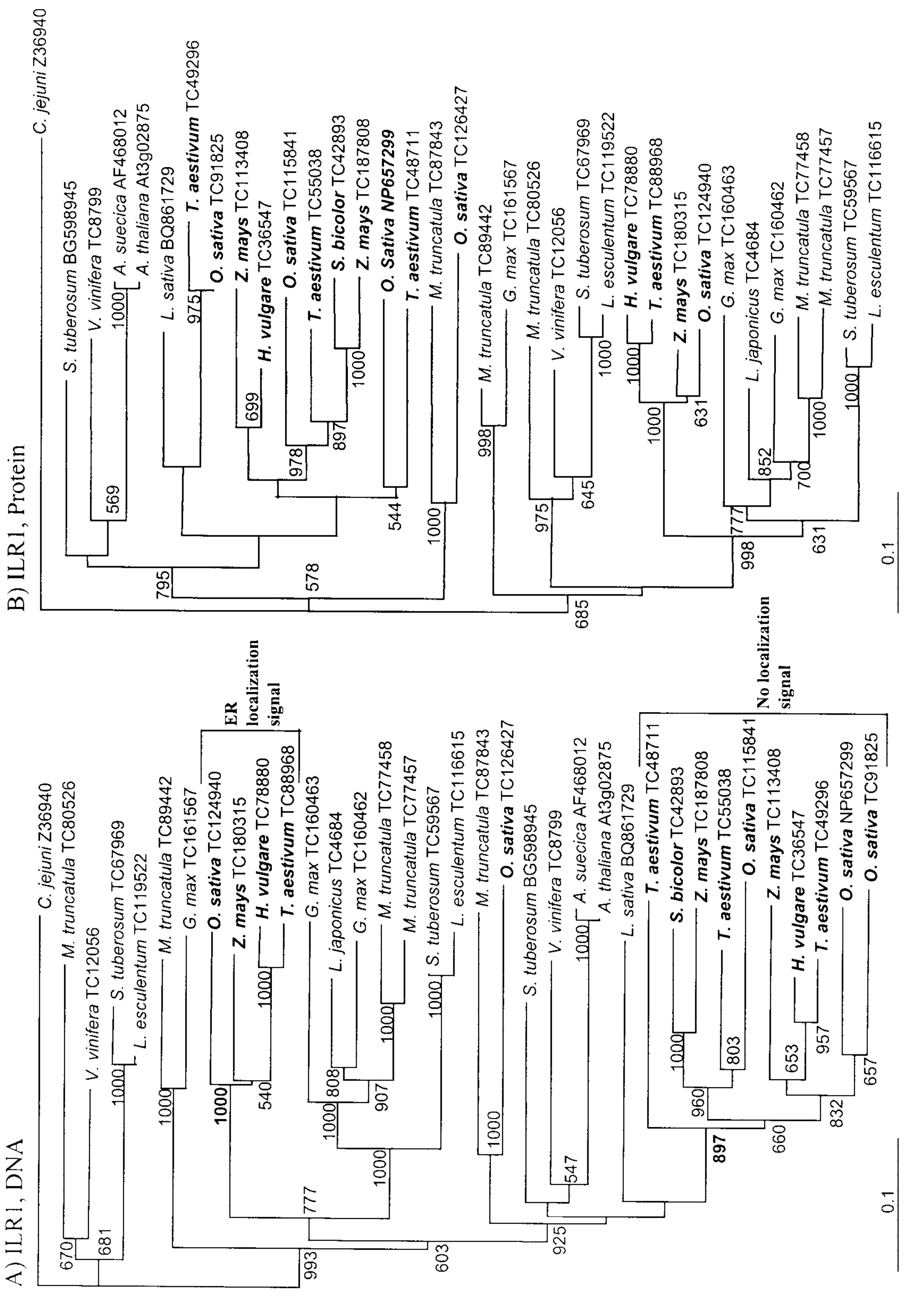

**Figure 1.** Neighbour-joining phylograms of species orthologous to *Arabidopsis* ILR1 and sILR1 genes. Monocotyledon species are in bold in both trees. (A) DNA sequence orthologue analysis. The bold bootstrap value indicates the high probability score for the predicted topology of the monocotyledon clades. (B) Protein sequence orthologue analysis

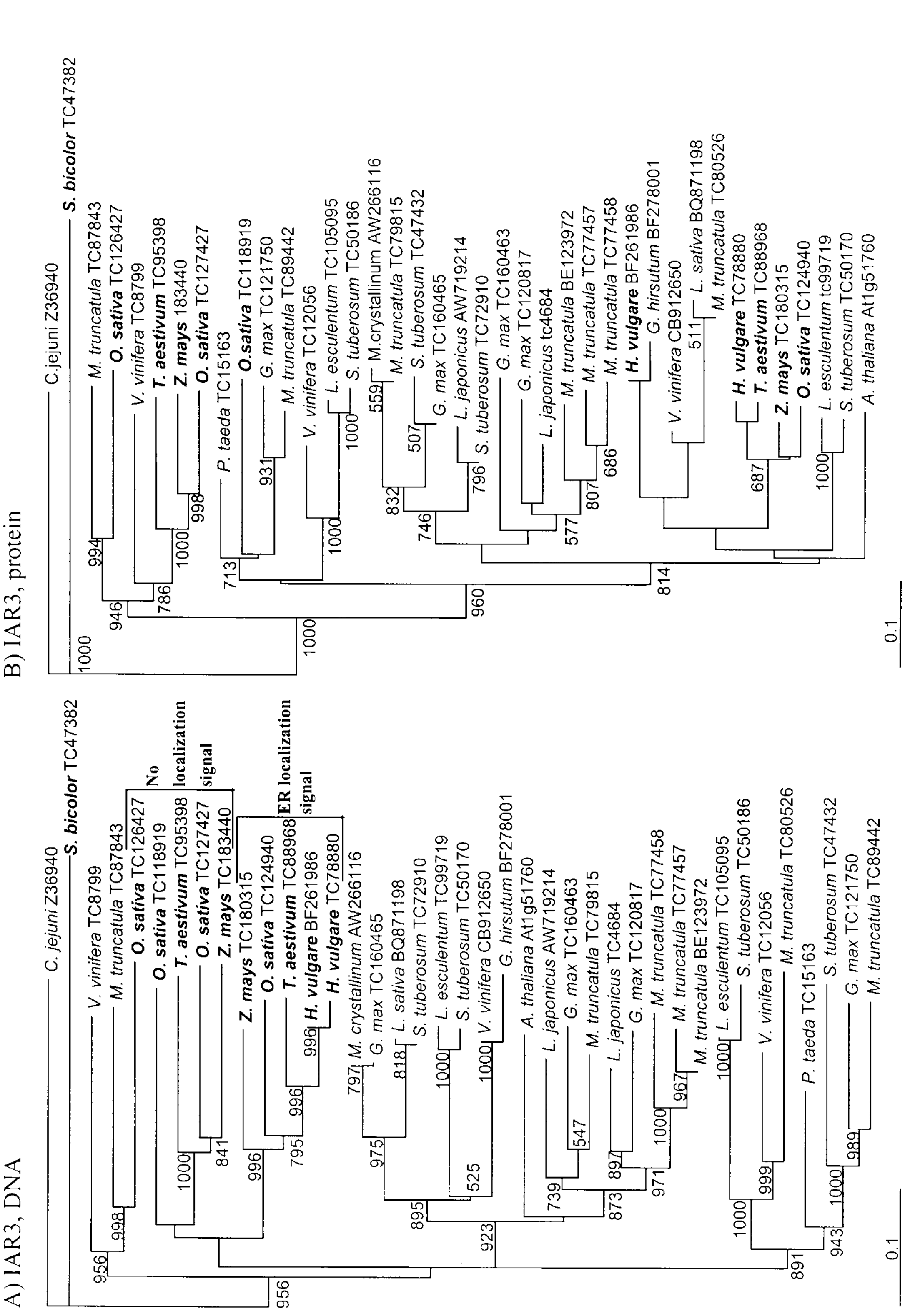

**Figure 2.** Neighbour-joining phylograms of species orthologous to the *Arabidopsis* IAR3 gene. Monocotyledon species are in bold in both trees. (A) DNA sequence orthologue analysis. (B) Protein sequence orthologue analysis sequence homology can be found so commonly. In addition to this interspecific conservation, the presence of multiple IAR3 orthologues in many species suggests that this gene has undergone intraspecific duplication (Figure 2).

As with ILR1, in the IAR3 trees there are two instances where tomato (TC105095, TC99719) and potato (TC50186, TC50170) orthologues appear in the same clade at both the DNA and protein levels (Figure 2).

Again, we see that the IAR3 monocotyledon orthologues, although sharing a common internode, have diverged into two distinct clades from the dicotyledons in the DNA tree (Figure 2A). The monocotyledon topology between IAR3's DNA and protein trees differs. The monocotyledon DNA sequence orthologues (Figure 2A), with the exception of the more distant sorghum sequence (TC47382), are grouped into two large clades with high conservation. The monocotyledon clades are split farther apart in the protein phylogram (Figure 2B), but the bulk of the orthologues parallel each other in topology (Figure 2).

Further evidence that IAR3 is highly conserved across species is that the pine orthologue (TC15163) does not appear as an outgroup in the IAR3 analyses. Note in IAR3 that the pine is a member of a clade with clover and soybean in both the DNA and protein trees (Figure 2).

ILL1 and IAR3 share tree topology to some degree (Figure 3) and reveal several of the same characteristics. The two homologues share 19 of the same orthologues in the same topological relationships (Table 1). Monocotyledon orthologues of ILL1, like IAR3, have again diverged into two close, but separate, clades (Figure 3A). A comparison of amino acid sequences indicates that ILL1 orthologue proteins also diverge into two separate clades, perhaps indicating functional differences (Figure 3B). Again, the Solanaceae species remain close to each other on the same branches.

In spite of this similarity to IAR3, ILL1 shares almost as many common orthologues (Table 1) with ILR1 (18), suggesting an equally high phylogenomic similarity to that gene, despite less topological correspondence in the phylograms (Figures 1, 3).

Previous literature (LeClere *et al.* 2002) has suggested that ILL1 and ILL2 are paralogues, i.e. duplicated homologues. LeClere *et al.* (2002) performed their studies using conserved hydrolase regions as probes. In our studies, we employed full-length cDNA of ILL1 and ILL2 to probe the sequence databases. We found evidence that the sequences are phylogenomically not as similar as has been previously indicated. ILL2 has a comparable number of overall orthologues (31) and orthologue-containing species (13) to ILL1 (Table 1). Of the 29 ILL2 orthologues, 20 are shared by ILL1, suggesting a less than perfect paralogy. In addition, the tree topology between the two homologues does not appear alike under the same conditions of analysis (Figures 3 and 4).

We found relatively few total orthologues for ILL6 (16) in the databases, while simultaneously uncovering a relatively large number of orthologous species (11). This may suggest that ILL6 has not been intraspecifically duplicated a great deal, but it still appears interspecifically conserved and has a relatively important physiological role. Again, we see in the ILL6 phylograms that monocotyledons have diverged on to a separate clade from dicotyledons (Figure 5). However, note that under stringent BLAST search conditions, ILL6 is unique in that it is homologous only to monocotyledon orthologues containing a putative ER localization signal.

The database searches with ILL3 sequence probes found the fewest orthologues (9) of the six ILR1-like family members analysed (Figure 6). This result, combined with ILL3 having the fewest orthologous species (7), suggests that ILL3 is perhaps the least conserved of the homologues examined. This finding may indicate that ILL3 has a less important physiological role in evolution than the other family members. However, all our phylogenomic analyses are ongoing and limited by the continually updated sequence databases, as well as the limitation of employing ESTs in sequencing studies.

ILL3 is homologous to the fewest monocotyledon orthologues (3) and none possess an ER localization signal (Figure 6). All three ILL3 monocotyledon orthologues (*Z. mays* TC136446, AY108 775; *T. aestivum* TC45334) are unique to ILL3 and do not distribute into the other monocotyledon clades (Figures 6 and 7). The only other monocotyledon sequence to show this characteristic is the rice gene TC126427, which consistently branches into a separated clade from the other monocotyledons (Figures 1, 3 and 7).

Copyright © 2003 John Wiley & Sons, Ltd. 

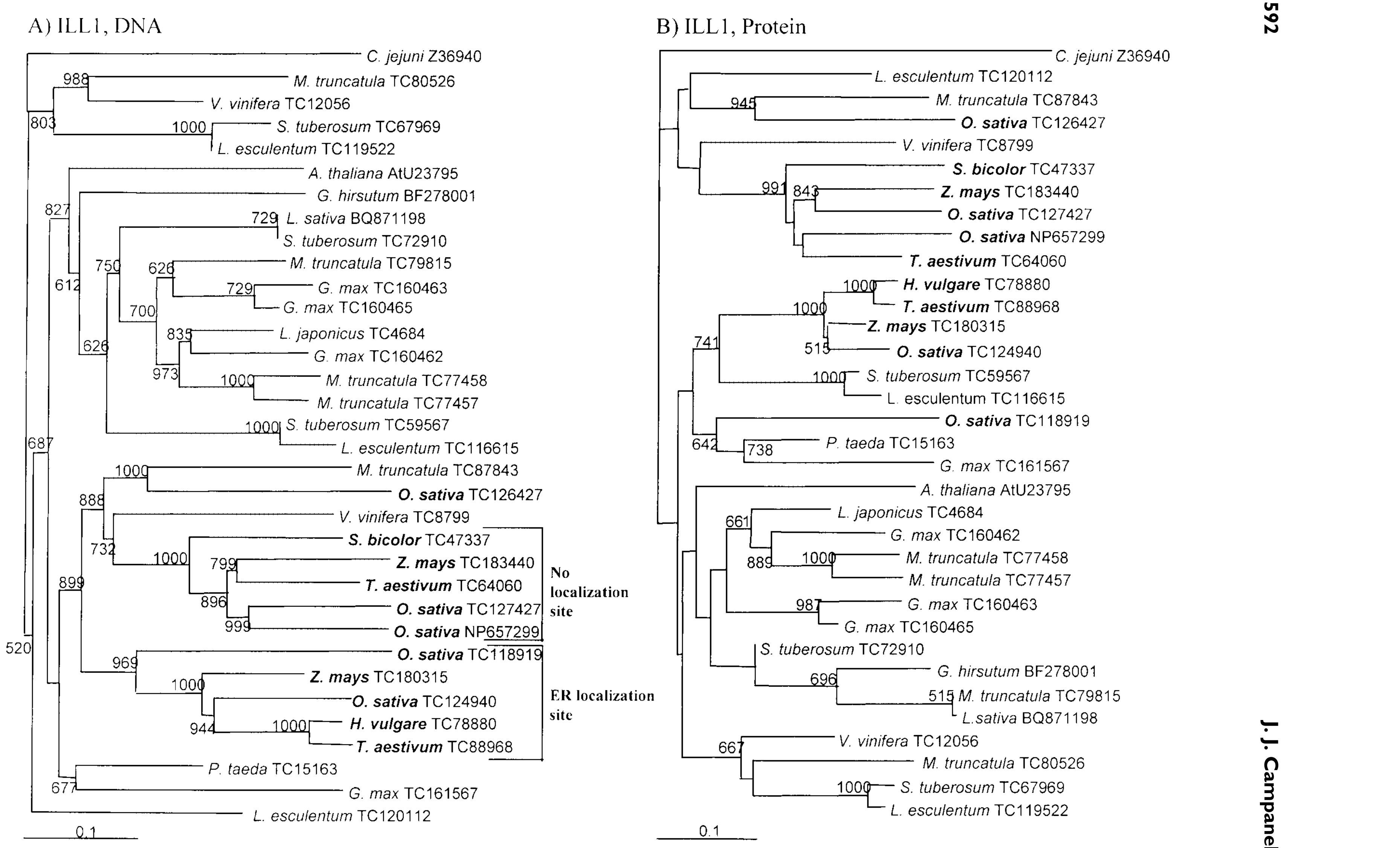

**Figure 3.** Neighbour-joining phylograms of species orthologous to the *Arabidopsis* ILL1 gene. Monocotyledon species are in bold in both trees. (A) DNA sequence orthologue analysis. (B) Protein sequence orthologue analysis

Copyright © 2003 John Wiley & Sons, Ltd.

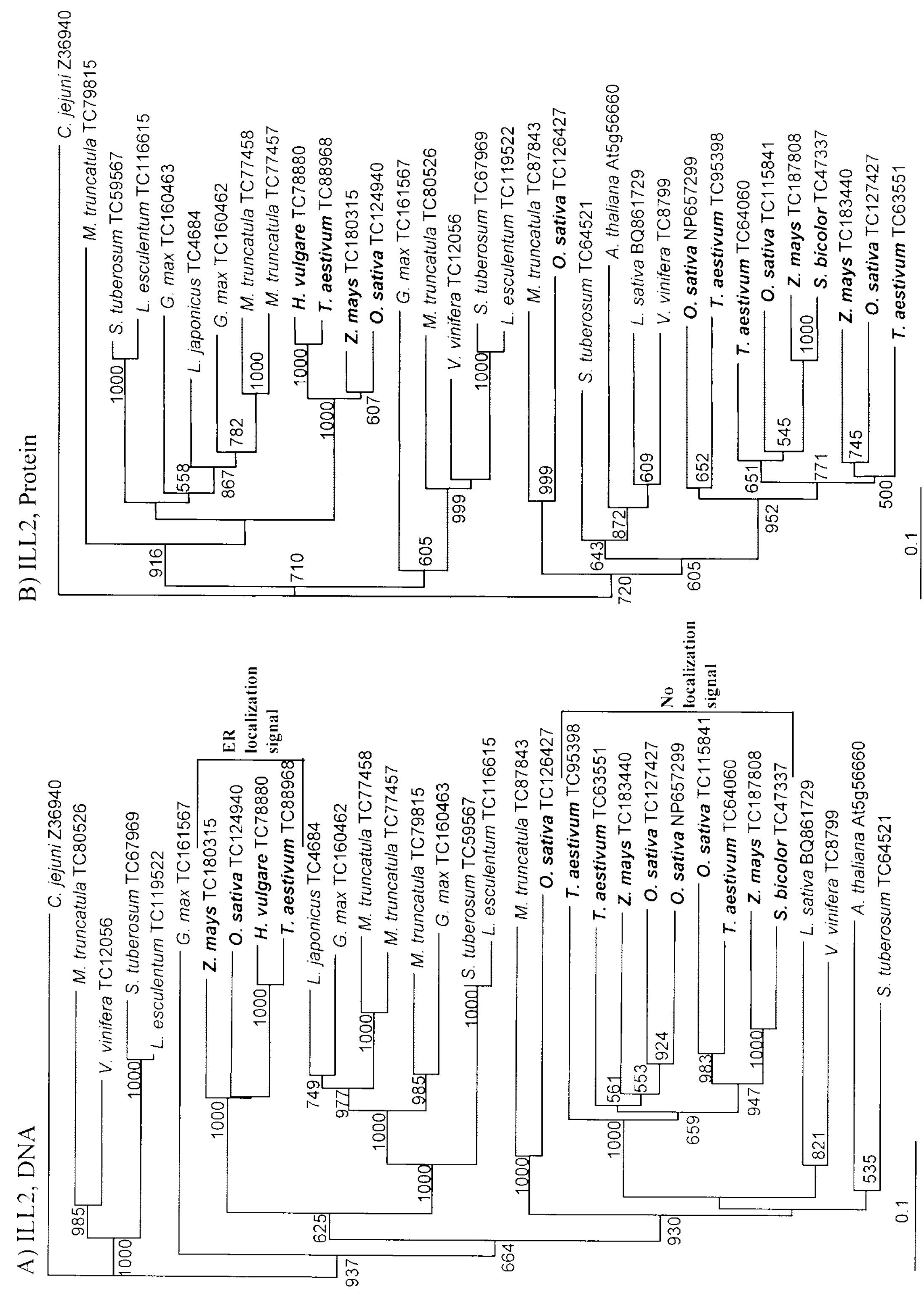

**Figure 4.** Neighbour-joining phylograms of species orthologous to the *Arabidopsis* ILL2 gene. Monocotyledon species are in bold in both trees. (A) DNA sequence orthologue analysis. (B) Protein sequence orthologue analysis

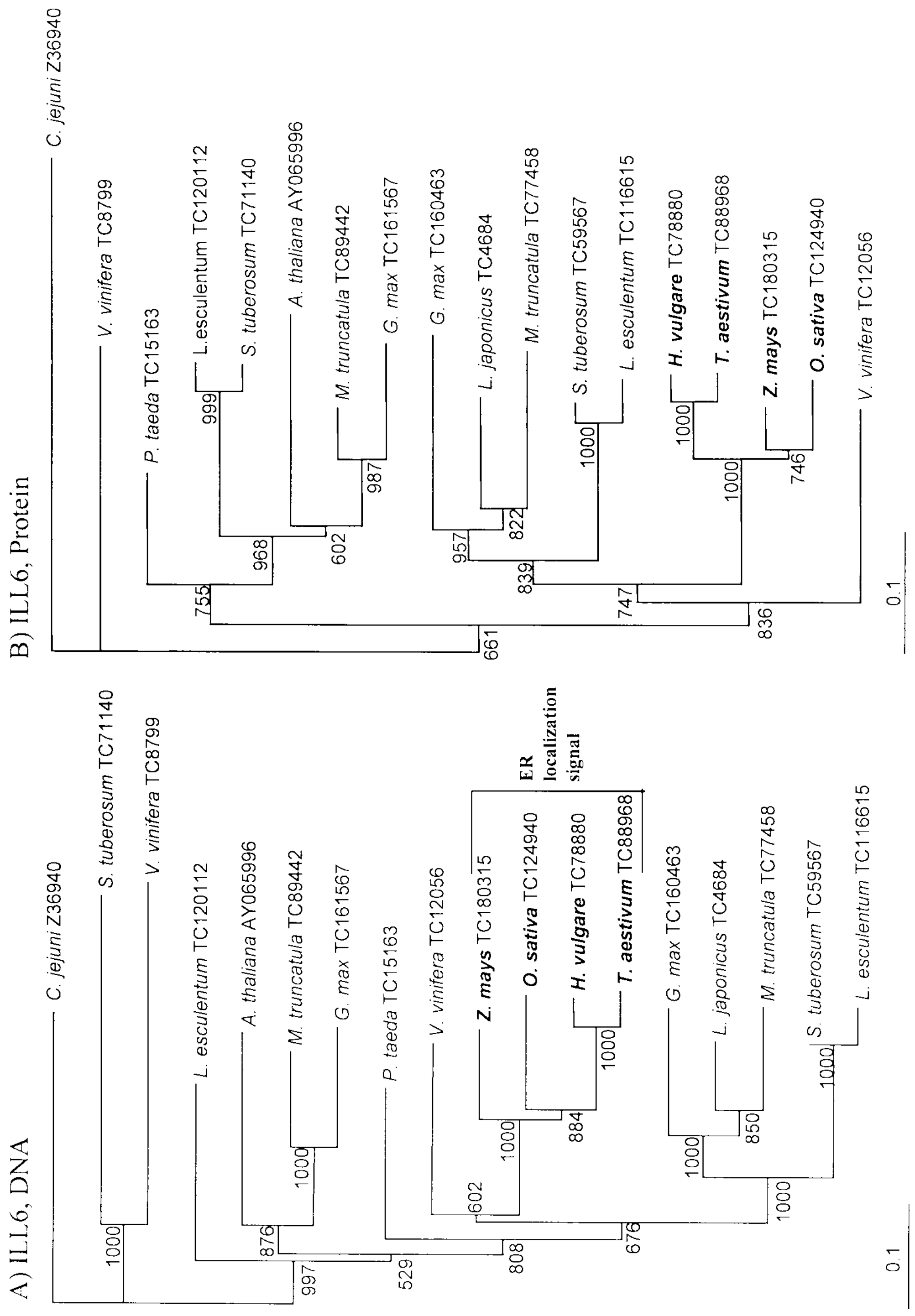

**Figure 5.** Neighbour-joining phylograms of species orthologous to the *Arabidopsis* ILL6 gene. Monocotyledon species are in bold in both trees. (A) DNA sequence orthologue analysis. (B) Protein sequence orthologue analysis

Copyright © 2003 John Wiley & Sons, Ltd. 

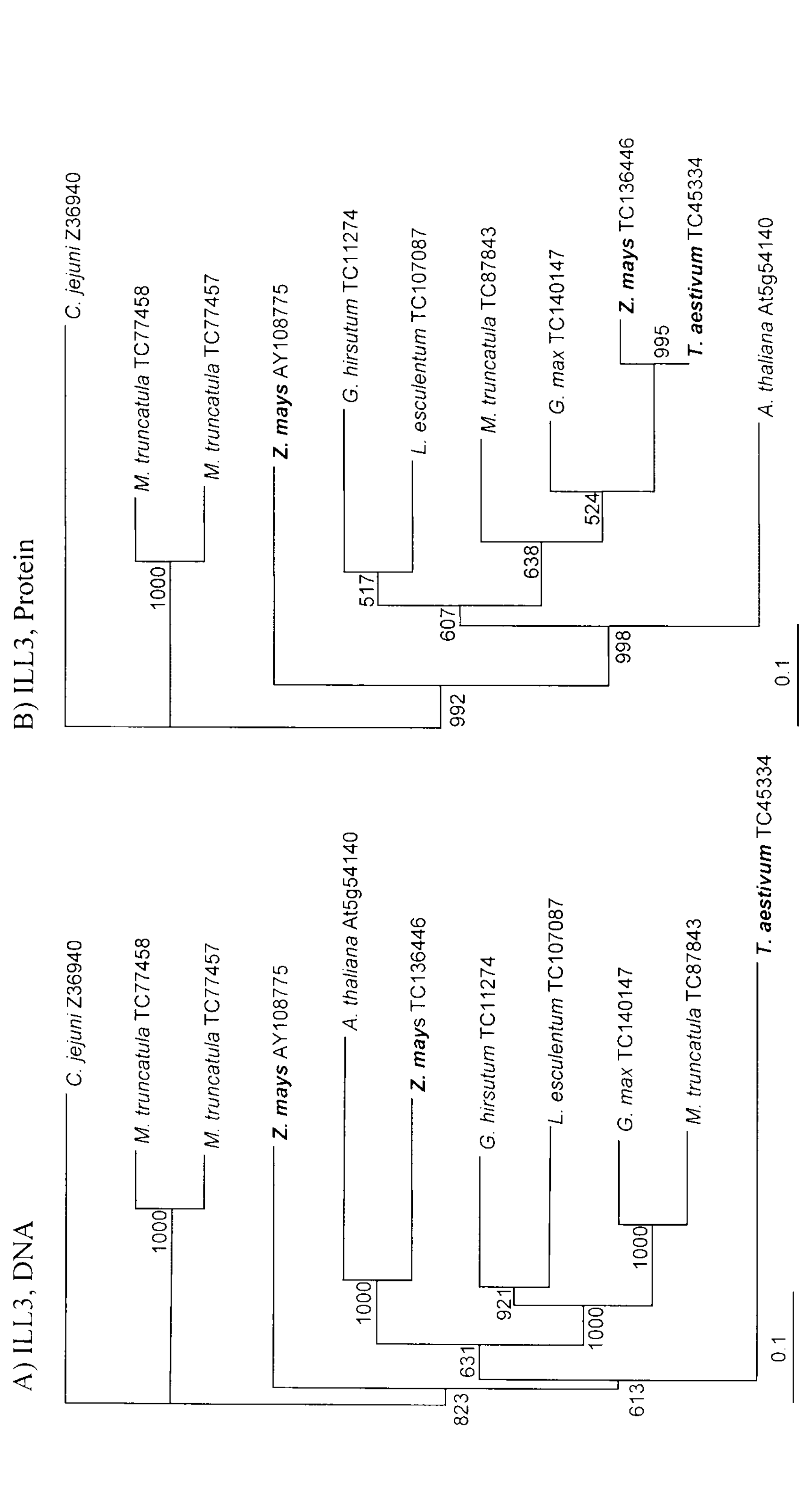

**Figure 6.** Neighbour-joining phylograms of species orthologous to the *Arabidopsis* ILL3 gene. Monocotyledon species are in bold in both trees. (A) DNA sequence orthologue analysis. (B) Protein sequence orthologue analysis

Copyright © 2003 John Wiley & Sons, Ltd. 

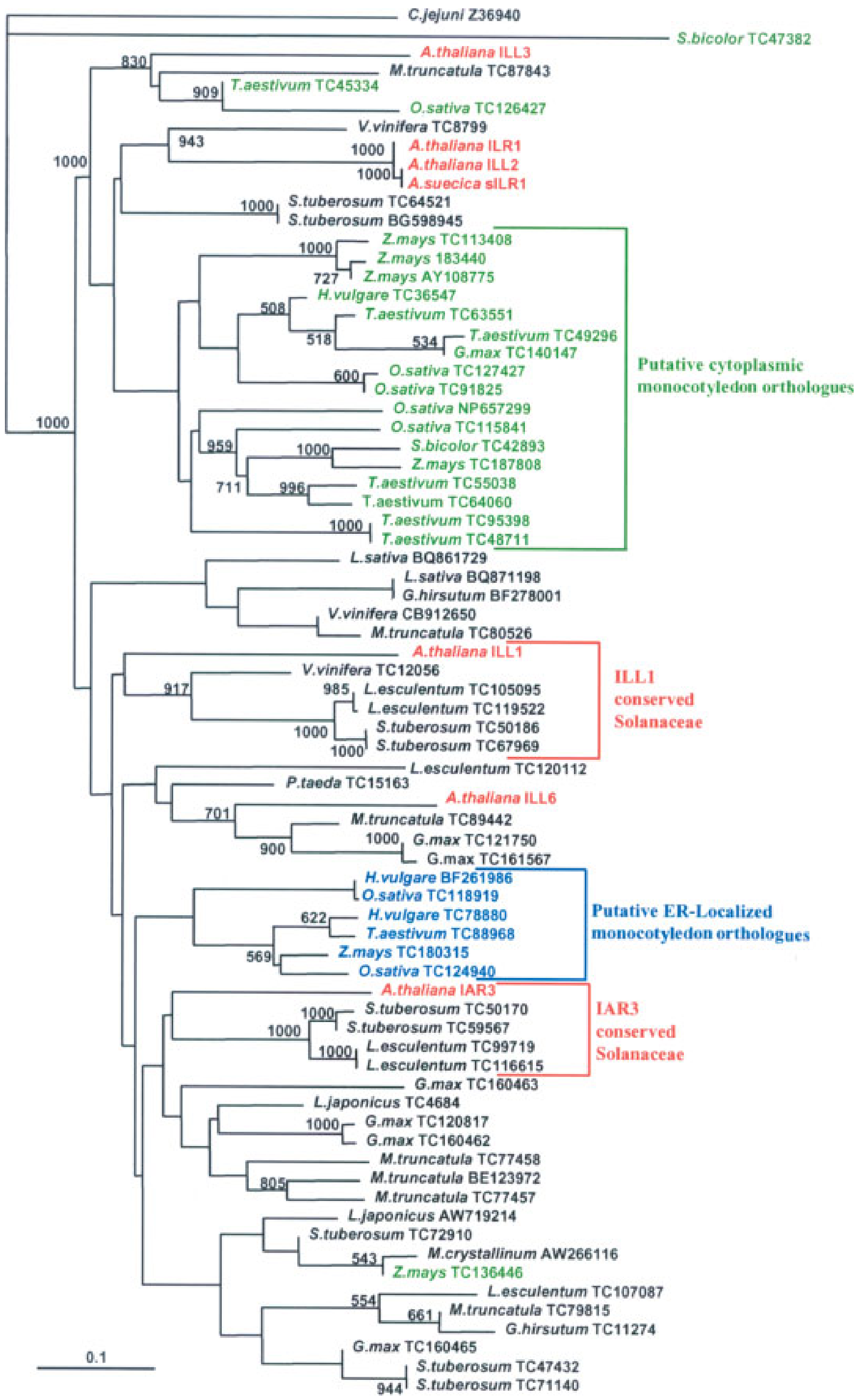

**Figure 7.** Neighbour-joining protein phylogram of all orthologues detected in the *Arabidopsis* ILR1-like gene family. Monocotyledon species with a putative cytoplasmic localization are coded in green. Monocotyledon species with a putative ER localization are coded in blue. Orthologues in red are members of the *A. thaliana* ILR1-like family. Solanaceae species diverge into two clades primarily related to ILL1 and IAR3

There are several monocotyledon orthologues that do not fall into a monocotyledon clade. The rice orthologue (TC126427) is one of these and can be seen in a clade with ILL3, along with the wheat orthologue (TC45334) in an overall protein phylogram (Figure 7). This rice orthologue possesses a complete coding sequence without an evident localization signal, so it is unclear why it does not cluster with other monocotyledons in phylograms in which it is present (Figures 1, 2, 3, 4 and 7). It is possible that its position on the various phylograms indicates a functional difference in its protein product. Another two isolated monocotyledon orthologues from corn (TC136446) and wheat (TC45334) are incomplete ESTs and as such not necessarily expected to cluster with the other monocotyledons (Figure 7). The last isolated monocotyledon is the semi-outgroup orthologue from sorghum (TC47382).

### Discussion

### ILR1-like family molecular evolution

There are several members of the ILRl-like family that have been isolated and analysed (Bartel and Fink, 1995; LeClere *et al.*, 2002; Davies *et al.*, 1999; Campanella *et al.*, 2003a, 2003c): ILL1, ILL2, ILL3, ILL5, ILL6, sILR1, ILR1, and IAR3. All the homologues were utilized in analyses presented here, except for ILL5. LeClere *et al.* (2002) have indicated that ILL5 is a duplicate of IAR3, as well as a pseudogene, so inclusion of this sequence in the study would have been redundant.

As earlier stated, it was also found by LeClere *et al.* (2002) that *Arabidopsis* ILL1 and ILL2 appear to be duplicated homologues. This conclusion was reached in performing a phylogenomic analysis employing only highly conserved regions of the ILR1-like family. Our own analysis using DNA global alignment with MatGAT v2.0 (Campanella *et al.* 2003b) indicates only an overall 50% identity between the sequences; therefore, we treated the two homologues separately in our analysis of the gene family. The ILL1 and ILL2 sequences, although they do show similarity in our studies (Figures 3 and 4), are not paralogous. They do not appear in the same clade in the overall protein phylogram analysis (Figure 7). ILL2 is in the same clade as ILR1 and sILR1, while ILL1 is quite distant in another clade with the conserved Solanaceae sequences (Figure 7). Note again that this interpretation is limited by the sequence databases, the sequencing process itself, and EST expression.

Based on the tree topology and total number of orthologues present, it appears that both the DNA and protein sequences for IAR3 have undergone fewer interspecific changes than the other ILR1-like family members, implying that IAR3 has been under strong selection for reduced variability. An additional piece of evidence supporting IAR3's strong conservation is that it possesses a majority of the unique orthologues that possess no other homologies (Table 1). Again, this apparent conservation supports the relative importance of IAR3 in the evolution of the physiological framework of 'higher' plant growth.

At the same time, we see a high level of intraspecific duplication in IAR3 sequences (Figure 2). Rice has four IAR3 orthologues, clover seven, soybean four, wheat two, barley two, grape three, potato four and tomato two. This duplication and evolutionary redundancy also supports the importance of the IAR3 gene. Barrel clover has undergone a higher number of IAR3 duplication events than the other species, and, perhaps, the duplication took place recently in evolutionary time, since a number of these genes are still in the same clade and have not yet diverged significantly from each other (Figure 7).

ILL1 and ILR1, based on their tree topology (Figures 1 and 3) and number of orthologues, appear to be almost as conserved as IAR3. Again, high numbers of interspecific copies and high levels of intraspecific duplication (Table 1) suggest that these two family members may share in the evolutionary and physiological importance of IAR3.

Monocotyledons are generally agreed to have diverged from dicotyledons 170–250 million years ago (Wolfe *et al.*, 1989; Martin *et al.*, 1993; Yang *et al.*, 1999; Kellogg, 2001). We know that IAR3, ILL1 and ILL6 genes (Table 1) must pre-date this point of divergence, since they are conserved not only in the angiosperms but in the gymnosperms as well. The gymnosperm–angiosperm divergence took place 300–360 million years ago, suggesting that the ILR1-like family is quite ancient and that its role is intimately tied to growth and development in 'higher' plants (Oliviusson *et al.*, 2001; Albert *et al.*, 2002; Cooke *et al.*, 2002).

Copyright © 2003 John Wiley & Sons, Ltd.

The ILR1-like family dates back to at least the divergence of the two major plant groupings.

Given that the ILR1-like family is so ancient, we examined databases of 'lower' plants to determine how far back in evolutionary time the family can be traced. However, a search of the Moss (*Physcomitrella*) EST Databases at University of California at Riverside (**http://138.23.191.152:/blast/blast.html**) and Leeds University, UK (**http://www.moss.leeds.ac.uk/blast.html**) showed no homologous sequences to any members of the ILR1-like family. Additional GenBank BLAST searches for ILR1-like orthologues in fern species also revealed no significant matches. We also tested *Chlamydomonas reinhardtii* in the TIGR database for orthologues but found none. Our lack of success in finding homologous sequences is most likely due to present limitations in the sequence databases of 'lower' plants.

It has been suggested that the presence of ILR1-like orthologues in bacterial species is direct evidence of the extremely ancient roots of this gene family (LeClere *et al.* 2002), but the case for this is rather ambiguous. Although the gene family is ancient, it seems that the IAA amidohydrolases may just resemble certain classes of bacterial hydrolases such as the hippuricases and aminoacylases (LeClere *et al.*, 2002). Many bacterial species express hydrolases that are members of the M20 peptidase family — of which the ILR1-like hydrolases are a sub-type — but these enzymes are present in many prokaryotic species. The conserved peptidase domains of these various hydrolases may be what are actually perceived as extremely ancient.

Conversely, the Cohen laboratory (Chou *et al.*, 1998) was able to isolate an active IAA-aspartate hydrolase from the bacterium *Enterobacter agglomerans.* The bacterial enzyme has a 20% homology to the ILR1-like family. With such a low sequence homology, these plant hydrolase enzymes may have resulted from convergent evolution.

There is literature support that plant IAA amidohydrolases may have arisen from convergent evolution and not from bacteria. Cooke *et al.* (2002) point out that IAA was an ancient signalling molecule in photosynthetic aquatic organisms, but that it seems unlikely that the IAA response system would have functioned in unicellular organisms prior to the major prokaryotic/eukaryotic evolutionary divergence over a billion years ago.

### Functional genomics of ILR1-like family

One physiological question that arises from the apparent high conservation of ILL1, ILR1 and IAR3 is what the actual functional role of these genes may be in monocotyledon and gymnosperm growth. Monocotyledons and gymnosperms primarily store IAA conjugated to sugars by ester bonds. The two orthologues ILR1 and sILR1, isolated from *A. thaliana* and *A. suecica,* have high activity against IAA conjugates bound to amino acids but no activity at all against sugar-bound conjugate substrates (LeClere *et al.*, 2002; Campanella *et al.*, 2003c). What is the substrate specificity of the ILR1-like monocotyledon and gymnosperm orthologues? Are these uncharacterized orthologues able to cleave amino acid-conjugates, sugar-conjugates or neither? Do these enzymes harbour some other substrate specificity?

Evolutionary change seems inevitable as orthologues in species more distant from *A. thaliana* are examined, but even closely related orthologues such as ILR1 and sILR1 have different substrate specificities.

ILR1 has low activity against IAA-glycine, whereas sILR1 is highly effective against the same substrate (Campanella *et al.*, 2003c). Additionally, sILR1 cannot cleave IAA-leucine, while ILR1 can do so (Campanella *et al.*, 2003c). This result indicates divergence over a relatively short evolutionary time span, since the point when *A. thaliana* and *Arabidopsis arenosa* hybridized to produce *A. suecica* (O'Kane *et al.* 1996).

Even greater functional changes are expected as phylogenetic distances get larger. We have already observed this phenomenon to be the case for at least one orthologue in one monocotyledon species. We have cloned and begun enzymatic characterization of a wheat IAR3 orthologue that we have dubbed TaIAR3 (J. J. Campanella, J. Ludwig-Mueller and A. Olajide, unpublished data). TaIAR3 possesses no substrate specificity for any of the IAA amino acid conjugates so far tested, although preliminary data suggests unique substrate recognition qualities. Characterization of this enzyme continues, but it is evident that the function of the monocotyledon orthologues of the ILR1-like family may differ substantially, since the monocotyledons diverge so clearly into separate clades (Figure 7).

These clades may reflect variations in enzymatic activity. We may speculate that the putatively

Copyright © 2003 John Wiley & Sons, Ltd.

ER-localized and cytoplasmically-localized monocotyledon hydrolases may differ functionally, since they are phylogenomically separated, but more enzymatic characterization is required to determine whether this is the case. It will be interesting to clone and characterize ILR1-like genes from gymnosperms and additional monocotyledons. We would like to determine whether our computer-based predictions for functional homology are validated.

### Acknowledgements

The authors wish to thank Dr Scott Kight for his thoughtful suggestions on this manuscript. We also wish to thank Lisa Campanella for her generous help in editing this article. This research was supported by a Sokol Grant for Undergraduate Research at MSU.

### References

Albert VA, Oppenheimer DG, Lindqvist C. 2002. Pleiotropy, redundancy and the evolution of flowers. *Trends Plant Sci* **7**(7): 297–299.

Altschul SF, Madden T, Schaffer A, *et al*. 1997. Gapped BLAST and PSI-BLAST: a new generation of protein database search programs. *Nucleic Acids Res* **25**: 3389–3402.

Andersson B, Sandberg G. 1982. Identification of endogenous N-(3-indoleacetyl)aspartic acid in Scots pine by combined gas chromatography–mass spectrometry, using high-performance liquid chromatography for quantification. *J Chromatogr* **238**: 151–156.

Bandurski RS, Ueda M, Nicholls PB. 1969. Esters of indole-3-acetic acid and myo-inositol. *Ann NY Acad Sci* **165**: 655–667.

Bandurski RS, Schulze A. 1977. Concentrations of IAA and its derivatives in plants. *Plant Physiol* **60**: 211–213.

Bartel B, Fink G. 1995. ILR1, an amidohydrolase that releases active indole-3-acetic acid from conjugates. *Science* **268**: 1745–1748.

Campanella J, Ludwig-Mueller J, Town CD. 1996. Isolation and characterization of mutants of *Arabidopsis thaliana* with increased resistance to growth inhibition by IAA-conjugates. *Plant Physiol* **112**: 735–745.

Campanella J, Bakllamaja V, Restieri T, *et al*. 2003a. Isolation of an ILR1 auxin hydrolase conjugate homologue from *Arabidopsis suecica*. *Plant Growth Regul* **39**: 175–181.

Campanella J, Bitincka L, Smalley J. 2003b. MatGAT: an application that generates similarity/identity matrices using protein or DNA sequences. *BMC Bioinformatics* **4**: 29.

Campanella JJ, Ludwig-Mueller J, Bakllamaja V, Sharma V, Cartier A. 2003c. ILR1 and sILR1 IAA amidohydrolase homologues differ in expression pattern and substrate specificity. *Plant Growth Regul* (in press).

Chou JC, Mulbury WW, Cohen JD. 1998. The gene for indole-3-acetyl-L-aspartic acid hydrolase from *Enterobacter agglomerans*: molecular cloning, nucleotide sequence, and expression in *Escherichia coli*. *Mol Gen Genet* **259**: 172–178.

Cohen JD. 1982. Identification and quantitative analysis of indole-3-acetyl-L-aspartate from seeds of *Glycine Max L. Plant Physiol* **70**: 749–753.

Cohen JD, Bandurski RS. 1982. The chemistry and physiology of the bound auxins. *Ann Rev Plant Physiol* **33**: 403–430.

Cooke TJ, Poli DB, Sztein AE, Cohen JD. 2002. Evolutionary patterns in auxin action. *Plant Mol Biol* **49**: 319–338.

Davies RT, Goetz DH, Lasswell J, Anderson MN, Bartel B. 1999. IAR3 encodes an auxin conjugate hydrolase from *Arabidopsis*. *Plant Cell* **11**(3): 365–376.

Domagalski W, Schulze A, Bandurski RS. 1987. Isolation and characterization of esters of indole-3-acetic acid from liquid endosperm of horse chestnut. *Plant Physiol* **84**: 1107–1113.

Felsenstein J. 1985. Confidence limits on phylogenies: an approach using the bootstrap. *Evolution* **39**: 783–791.

Genemark. DNA analysis programs. 2003. **http://opal.biology.gatech.edu/GeneMark/**.

Kellogg EA. 2001. Evolutionary history of the grasses. *Plant Physiol* **125**: 1198–1205.

Kopcewicz J, Ehmann A, Bandurski RS. 1974. Enzymatic esterification of IAA to myo-inositol and glucose. *Plant Physiol* **54**: 846–857.

Kowalczyk M, Sandberg G. 2001. Quantitative analysis of indole-3-acetic acid metabolites of *Arabidopsis*. *Plant Physiol* **127**(4): 1845–1853.

LeClere S, Tellez R, Rampey RA, Matsuda SP, Bartel B. 2002. Characterization of a family of IAA-amino acid conjugate hydrolases from *Arabidopsis*. *J Biol Chem* **277**: 20 446–20 452.

Ljung K, Ostin A, Lioussanne L, Sandberg G. 2001. Developmental regulation of indole-3-acetic acid turnover in Scots pine seedlings. *Plant Physiol* **125**(1): 464–475.

Martin W, Lydiate D, Brinkmann H, *et al*. 1993. Molecular phylogenies in angiosperm evolution. *Mol Bio Evol* **10**: 140–162.

Moss EST Database BLAST, University of California at Riverside. 2003. **http://138.23.191.152:/blast/blast.html**.

Moss EST Database BLAST, Leeds University. 2003. **http://www.moss.leeds.ac.uk/blast.html**.

O'Kane SL, Schall BA, Al-Shehbaz IA. 1996. The origins of *Arabidopsis suecica* (Brassicaceae) as indicated by nuclear rDNA sequences. *Syst Bot* **21**: 559–566.

Oliviusson P, Salaj J, Hakman I. 2001. Expression pattern of transcripts encoding water channel-like proteins in Norway spruce. *Plant Mol Biol* **46**(3): 289–299.

Ostin A, Moritz T, Sandberg G. 1992. Liquid chromatography/mass spectrometry of conjugates and oxidative metabolites of indole-3-acetic acid. *Biol Mass Spectrom* **21**: 292–298.

Page RD. 1996. TREEVIEW: an application to display phylogenetic trees on personal computers. *Comput Applic Biosci* **12**: 357–358.

Rice P, Longden I, Bleasby A. 2000. EMBOSS: the European molecular biology open software suite. *Trends Genet* **16**(6): 276–277.

Saitou N, Nei M. 1987. The neighbour-joining method: a new method for reconstructing phylogenetic trees. *Mol Biol Evol* **4**: 406–425.

Sandberg G. 1984. Biosynthesis and metabolism of indole-3-ethanol and indole-3-acetic acid by *Pinus sylvestris* needles. *Planta* **161**: 398–403.

Copyright © 2003 John Wiley & Sons, Ltd. 

Sonner JM, Purvis WK. 1985. Natural occurrence of indole-3-acetyl-aspartate and indole-3-acetyl-glutamate in cucumber shoot tissue. *Plant Physiol* **77**: 784–785.

Tam YY, Epstein E, Normanly J. 2000. Characterization of auxin conjugates in *Arabidopsis*. Low steady-state levels of indole-3-acetyl-aspartate, indole-3-acetyl glutamate, and indole-3-acetyl-glucose. *Plant Physiol* **123**(2): 589–596.

The Institute for Genomic Research database BLAST search site. 2003. **http://tigrblast.tigr.org/tgi**.

Thompson JD, Gibson TJ, Plewniak F, *et al*. 1997. The Clustal X windows interface: flexible strategies for multiple sequence alignment aided by quality analysis tools. *Nucleic Acids Res* **24**: 4872–4882.

Wolfe KH, Gouy M, Yang YW, *et al*. 1989. Date of the monocotyledon-dicotyledon divergence estimated from chloroplast DNA sequence data. *Proc Natl Acad Sci USA* **86**: 6201–6205.

Yang YK, Lai KN, Tai PY, Li WH. 1999. Rates of nucleotide substitution in angiosperm and mitochondrial DNA sequences and dates of divergence between *Brassica* and other angiosperm lineages. *J Mol Evol* **48**(5): 597–604.

Copyright © 2003 John Wiley & Sons, Ltd. 
